# Laparoscopic orchiopexy of palpable undescended testes_ experience of a single tertiary institution with over 773 cases

**DOI:** 10.1186/s12887-020-2021-6

**Published:** 2020-03-16

**Authors:** Jia You, Gang Li, Haitao Chen, Jun Wang, Shuang Li

**Affiliations:** Department of Pediatric Urology Surgery, Wuhan Children’s Hospital (Wuhan Maternal and Child Healthcare Hospital), Tongji Medical College, Huazhong University of Science and Technology, No.100, Hong Kong Road, Jiang’an District, Wuhan, 430016 China

**Keywords:** Laparoscopy, Inguinal, Cryptorchidism, Palpable, Undescended testes, Orchiopexy

## Abstract

**Background:**

Discuss the superiority of laparoscopic orchiopexy in the treatment of inguinal palpable undescended testes.

**Methods:**

Inclusion criteria: Preoperative examination and color Doppler ultrasound examination confirmed that the testes were located in the inguinal canal and could not be pulled into the scrotum, except for retractive and ectopic testes. The surgical steps were depicted as follow. The retroperitoneal wall was carved by ultrasonic scalpels, separates the spermatic vessels closed to the inferior pole of the kidney if necessary, dissects the peritoneum of vas deferens, cuts the testicular gubernaculum, and pulls back the testicle into the abdominal cavity. Besides, protect the vas deferens, and descend the testes to the scrotum and fix them without tension.

**Results:**

There were 773 patients with 869 inguinal undescended palpable testes, 218 cases on the left side, 459 cases on the right side and 96 cases with bilateral undescended testes, whose age ranged from 6 months to 8 years, with an average of 20 months. All testes were successfully operated, no converted to open surgery. The average operation time was (34.8 ± 5.4) min. There were 692 testes have an ipsilateral patent processus vaginalis (89.5%); In 677 cases of unilateral cryptorchidism, 233 cases (34.4%) have a contralateral patent processus vaginalis, and laparoscopic percutaneous extraperitoneal closure the hernia sac carry out during the surgery. There was no subcutaneous emphysema during the operation, no vomiting, no abdominal distension, no wound bleeding and obvious pain after surgery, especially wound infection is rarely. Doppler ultrasound was evaluated regularly after surgery. The patients were followed up for 6 to 18 months. All the testes were located in the scrotum without testicular retraction and atrophy. No inguinal hernia or hydrocele was found in follow-up examination.

**Conclusion:**

Laparoscopic orchiopexy manage inguinal palpable cryptorchidism is safe and effective, and there are obvious minimally invasive advantages. Furthermore, It could discover a contralateral patent processus vaginalis, and treat at the same time, which avoid the occurrence of metachronous inguinal hernia.

## Background

Cryptorchidism, or undescended testes, is not uncommon in children’s congenital malformations. This condition usually involves the testicle failing to move from the abdomen through the inguinal canal to the scrotum during fetal development. According to the position of the testis, the cryptorchidism usually classified to inguinal cryptorchidism and intra-abdominal cryptorchidism in clinical practice, and which above 80% of cryptorchidism can touch the testis in the groin area. Cryptorchidism over 6 months needs to be fixed in the scrotum by surgery. Currently, laparoscopy has been widely used to diagnose and treat intra-abdominal cryptorchidism, while it is still controversial for the treatment of inguinal cryptorchidism that can touch the testis [1], the classical surgical approach is trans-inguinal orchiopexy, however, there are some flaws in this surgical procedure [2–4].

Since Decimo et al. [5] firstly introduced laparoscopic orchiopexy for the high palpable undescended testis, some studies [6, 7] began to explore laparoscopy for treatment of inguinal cryptorchidism, pointed out that the laparoscopic technique is safe and feasible, however, no large amount of cases have been reported. In addition, It is well known that inguinal hernia is a common concomitant complication of cryptorchidism, the research [8, 9] reported approximately 64–92% of cryptorchidism with ipsilateral patent processus vaginalis, nevertheless, fewer literature [9] focus on the relationship between inguinal palpable cryptorchidism with contralateral patent processus vaginalis, which could develop into metachronous inguinal hernia.

The paramount purpose of this study was to discuss the superiority of laparoscopic orchiopexy in the treatment of inguinal palpable undescended testes, secondly, confirm the incidence of inguinal palpable testis associated with a contralateral patent processus vaginalis discovered during laparoscopy. We retrospective analyze the recorded and collected data from January 2012 to December 2017 for the laparoscopic treatment of 773 cases of inguinal cryptorchidism, to the best of our knowledge, this is the largest cases in published research so far.

## Methods

### Inclusion criteria

Preoperative examination and color Doppler ultrasound examination confirmed that the testes were located in the inguinal canal and could not be pulled into the scrotum, except for retractive and ectopic testes. A total of 773 cases of 869 testes were included in the study, 218 (28.2%) on the left, 459 (59.4%) on the right, and 96 (12.4%) on both sides, aged 6 months to 8 years, with an average of 20 months.

### The surgical method

**Fig. 1:**
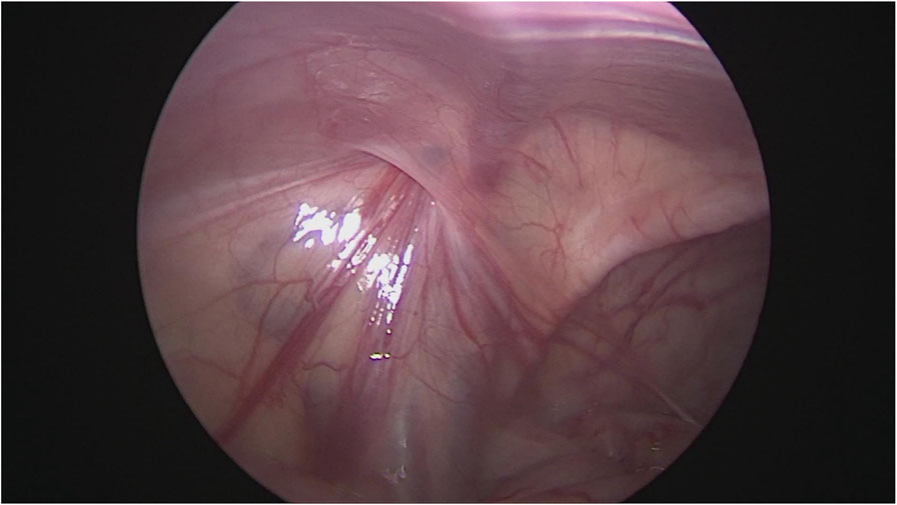
Laparoscopic view of a left patent contralateral processus vaginalis

**Fig. 2:**
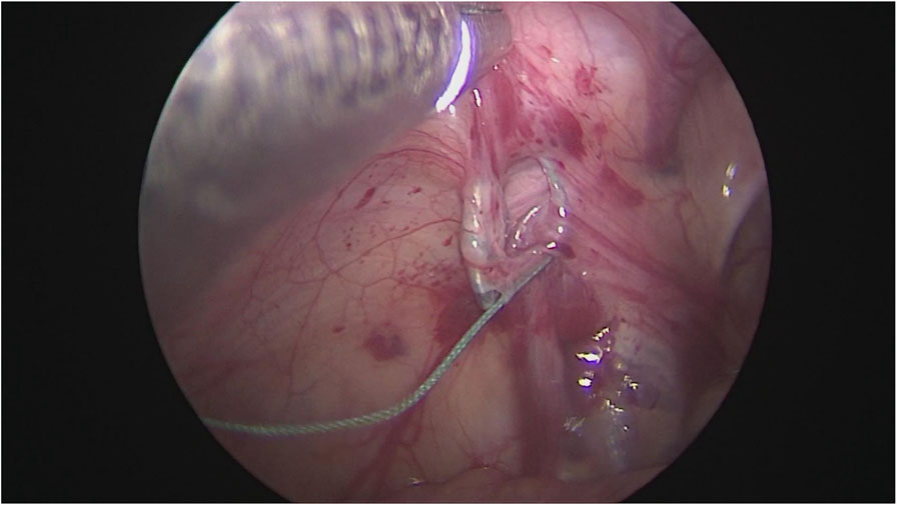
Closure of the hernia sac under Laparoscopically assisted

**Fig. 3:**
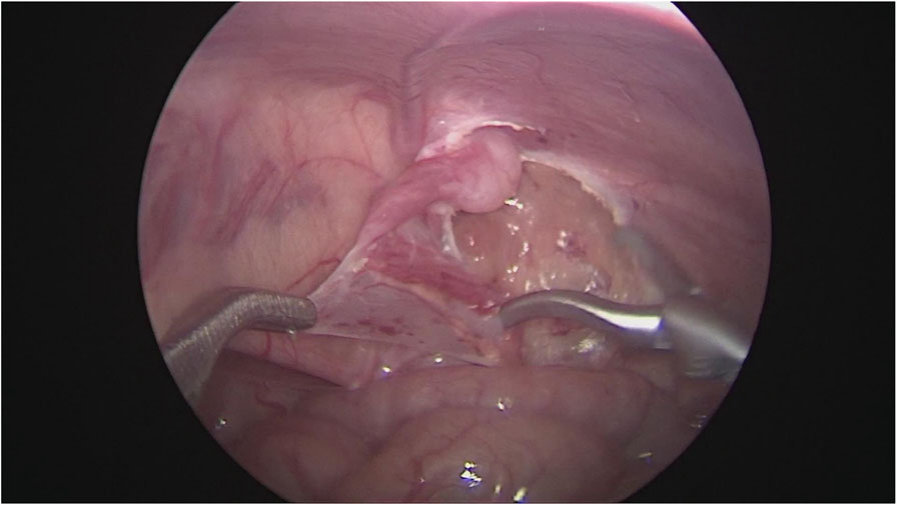
Separation of spermatic vessels

**Fig. 4:**
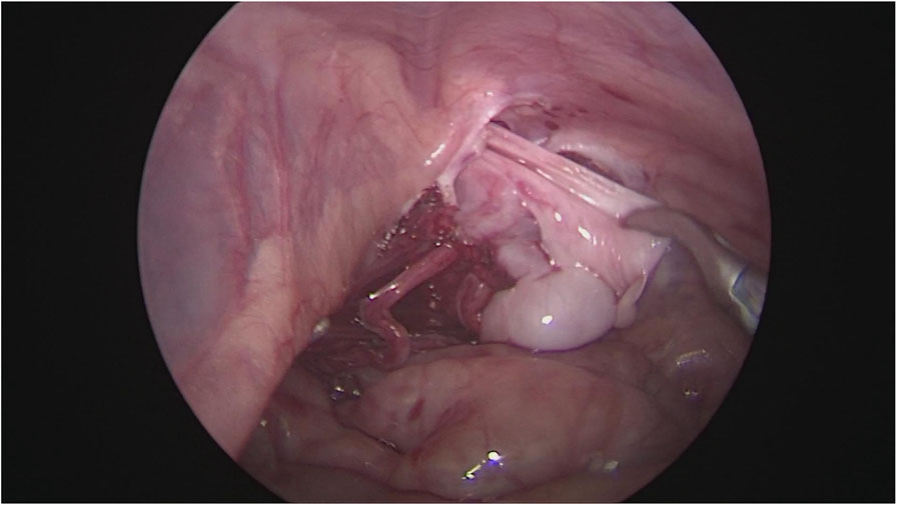
Free and protection of the vas deferens

**Fig. 5:**
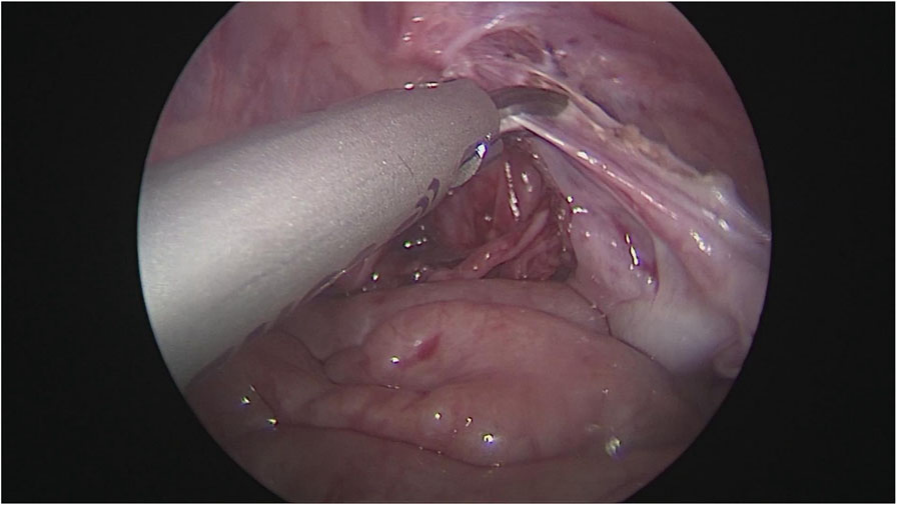
Cut off the testicular gubernaculum under visualization

**Fig. 6:**
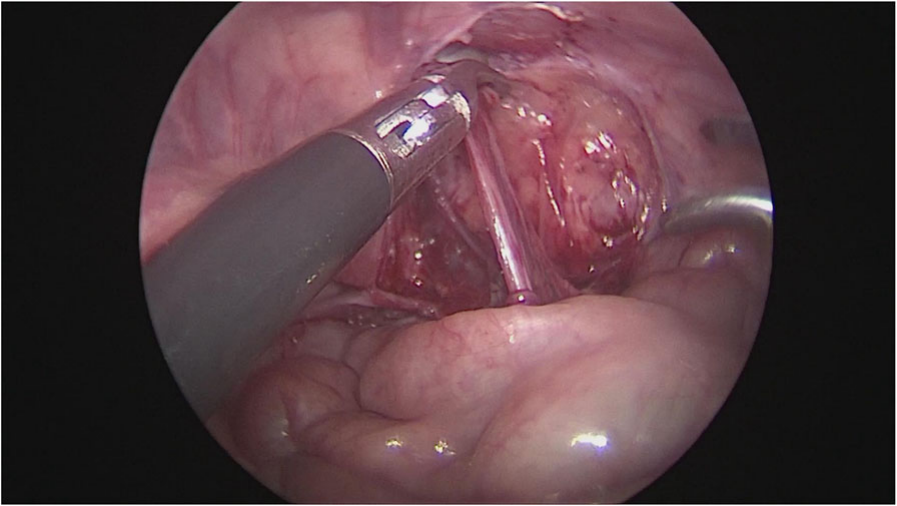
Free spermatic vessels to the inferior pole of the kidney when necessary

After general anesthesia, supine position, take the lower edge of the umbilicus, the lateral margin of the rectus abdominis and the small incision of the lower abdomen, about 0.5 cm long, put 5 mm Trocar, establish pneumoperitoneum (8-10 mmHg), laparoscopic exploration of the abdominal cavity. There is no testicle inside abdominal, spermatic cord and vas deferens enter along the inguinal region from the internal ring, and the spermatic vessels are finely (Fig. 1). Cutting the peritoneal posterior wall, dissociating the spermatic cord closed to the inferior pole of kidney when it is necessary, and separating the vas deferens from the posterior peritoneum. Free the adhesion of the spermatic cord in the inguinal canal, cut off the testicular gubernaculum, pull the testicle back into the abdominal cavity, protect the vas deferens, use the home-made guide device to pass through the testes from inguinal internal ring descend to the scrotum, and fix in the scrotum sac. During the operation, the ultrasonic scalpel is used to dissection the peritoneum at the internal ring instead of closed, and, if associated with a contralateral patent processus vaginalis, the occult inguinal hernia sac would be performed percutaneous extraperitoneal closure in a same surgery (Figs. 2, 3, 4, 5 and 6).

## Results

All the testes were successfully performed with laparoscopic orchiopexy, none of the cases needed conversion to open surgery, the operation time was (34.8 ± 5.4) min. There was a patent processus vaginalis found in 692 cases of the affected side (89.5%), in 677 cases of unilateral cryptorchidism, a contralateral processus vaginalis was unclosed in 233 cases (34.4%), which was closed in the surgery as well. All children had no subcutaneous emphysema, and they were fed within 6 h after general anesthesia. There was no vomiting, abdominal distension, no wound bleeding and obvious pain. Color Doppler ultrasound was reviewed regularly after surgery. The patients were followed up for 6 to 18 months after surgery. The testes were examined, located in the scrotum without testicular retraction and atrophy. No inguinal hernia or hydrocele was found (Table 1).

**Table 1:** Patient characteristics with palpable inguinal undescended testes

Patient characteristics	Values
Birth weight (mean)	3.3 kg
Body weight (mean)	13.2 kg
Side	
unilateral	677 (87.6%)
bilateral	96 (12.4%)
Patent processus vaginalis	
ipsilateral	692 (89.5%)
contralateral	233 (34.4%)
Operation time	34.8 ± 5.4 min
Follower-up period	6–18 month
Ipsilateral inguinal hernia	76 (9.8%)

## Discussion

Laparoscopy used for the diagnosis and treatment of nonpalpable intra-abdominal cryptorchidism has been universally accepted in clinical practice, while the inguinal palpable cryptorchidism is tended to the traditional typical open inguinal orchiopexy [2]. However, there are some conspicuous defects in this surgical procedure; firstly, the conventional inguinal incision is not easy to be adequately exposed, and it is likely to damage the testicular blood supply when separating the retroperitoneal spermatic cord vessels, which increases the risk of postoperative testicular atrophy. Neheman et al. [3] reported 5 cases of testicular atrophy in 134 cases of inguinal cryptorchidism by trans-inguinal orchiopexy, the rate is about 3.7%. Ein et al. [4] indicated that testicular atrophy occurred in about 5% of low typical inguinal cryptorchidism, while the figure reach up to 9% in higher inguinal position when testicular fixation by inguinal open approach. So far, testicular atrophy has not been found in 773 patients in this group. Secondly, open surgery requires separate the inguinal canal, which not only destroys the anatomical structure of the inguinal canal, but also needs to cut the intra-abdominal oblique muscle and the transverse abdominis muscle at the inner ring for a higher position of the inguinal typical cryptorchidism, which would be prone to occur wound infection, bleeding, and even testicular retraction; according to the literature, the wound infection rate of open inguinal orchiopexy is 1.9–2.5% [2, 10]. At last, the most important is trans-inguinal orchiopexy unable to detect a contralateral occult hernia or patent processus vaginalis, cryptorchidism associate with a contralateral patent processus reach up to 33–40% [7, 9], while preoperative color Doppler ultrasound examination only discovers about 20% [11]. A prospective study in Japan indicated that the diameter of the contralateral processus vaginalis can be developed into symptomatic inguinal hernia with a diameter of > 2 mm, with specificity and sensitivity of 81.8 and 71.3%, respectively [12].

In 1995, Docimo et al. [5] first introduced laparoscopic orchiopexy for treatment of cryptorchidism that can touch the testicles in the inguinal canal. Subsequently, Mario et al. [6], He et al. [7] confirmed that the technology is feasible, safe and effective. Compared with traditional open surgery, laparoscopic technology has obvious advantages: above all, by the amplification of laparoscopy, it is easier to loosen and separate the retroperitoneal spermatic vessels under visualization, and even reach the proximal next to the inferior pole of the kidney when it is necessary. The testis is fixed in the scrotum without tension, which can effectively reduce the occurrence of testicular atrophy and retraction. Then the anatomical integrity of the inguinal canal is maintained. For the high inguinal typical cryptorchidism, the intra-abdominal oblique muscle and the transverse abdominis muscle can be avoided cutting at the inner ring. Compared with the traditional open surgical methods, the postoperative pain is smaller, and the recovery is faster. No obvious surgical scars, parents have higher satisfaction. Finally, laparoscopic surgery can simultaneously detect a contralateral occult hernia. Studies have shown that more than 10% of asymptomatic hernia accidentally found by laparoscopic will develop into symptomatic metachronous inguinal hernia [13, 14], which require surgery again under anesthesia. A 17-year follow-up study of Taiwan also pointed out that up to 12% of contralateral occult hernia can develop into clinically inguinal hernia, about 63% of the symptoms appear within 2 years after the affected side operated, and the ratio will be as high as 91% in 5 years [15]. Laparoscopic orchiopexy can simultaneously treat contralateral occult hernia, avoid suffering a second pain and fear caused by reoperation and anesthesia. The study reveals that 34% of palpable cryptorchidism associate with a contralateral patent processus vaginalis, consistent with the reported literature [9].

There are two remarkable limitations of our research. First, our follow-up period is too short to making an authoritative assessment of outcome after surgery for undescended testis. The second shortage of our research is that we are indeterminable that a contralateral patent processus vaginalis truly indicates that these children will develop into clinically inguinal hernia in the future. But what I can definitely believe is that it will significantly reduce the chance of suffering reoperation and anesthesia.

## Conclusions

About 34% of inguinal palpable undescended testes associated with a contralateral patent processus vaginalis. Laparoscopic orchiopexy for the treatment of inguinal cryptorchidism is safe and less invasive than the open groin surgery, and can simultaneously detect and treat contralateral occult hernia, which avoiding the occurrence of metachronous inguinal hernia.

## Data Availability

The raw dataset analyzed in the current study are available from the corresponding author on reasonable request.
